# Fusarium solani Necrotizing Fasciitis Complicating Treatment for Acute Lymphoblastic Leukemia: A Case Report

**DOI:** 10.7759/cureus.25847

**Published:** 2022-06-11

**Authors:** Fatma Al-Farsi, Abdullah Balkhair, Turkiya Al-Siyabi, Asim Qureshi

**Affiliations:** 1 Medical Microbiology Residency Programme, Oman Medical Specialty Board, Muscat, OMN; 2 Infectious Disease, Sultan Qaboos University Hospital, Muscat, OMN; 3 Microbiology and Immunology, Sultan Qaboos University Hospital, Muscat, OMN; 4 Pathology, Sultan Qaboos University Hospital, Muscat, OMN

**Keywords:** azoles, leukemia, amphotericin, fungal infections, fusarium

## Abstract

Fungal infections due to *Fusarium *species are serious albeit rare and mostly occur in severely immunocompromised patients. The prognosis of such infections, especially of disseminated manifestations, is poor as a result of multi-antifungal resistance, particularly to azoles. We report a case of a rapidly progressive necrotizing fasciitis of the foot secondary to Fusarium solani in a young female patient with acute lymphoblastic leukemia on consolidation therapy. Surgical debridement was undertaken and liposomal amphotericin was given as definitive therapy for a total of six weeks followed by secondary prophylaxis that resulted in remarked clinical and radiological improvement. High clinical suspicion, prompt surgical intervention, rapid diagnosis, and timely initiation of appropriate antifungal therapy are crucial for a favorable outcome in this relatively uncommon life-threatening infection.

## Introduction

Fusarium species are emerging opportunistic fungi in immunocompromised patients with infections ranging from superficial to locally invasive and disseminated disease [1]. Fusarium is arguably the second most common mold infection in immunocompromised patients with an exceedingly high mortality rate [2]. Fusarium species exhibit global distribution in the environment. Fusariosis may potentially be acquired by the inhalation of airborne Fusarium conidia, direct inoculation into the skin or mucous membrane following trauma or the host’s own gut is considered as a source of infection, which is regularly colonized with Fusarium species [3,4]. Prolonged neutropenia or severe T-cell immunodeficiency poses a major risk for fusariosis [3]. Disseminated fusariosis accounts for 56% of Fusarium infections in acute leukemia patients [5]. Early identification and appropriate selection of antifungal agents are key to successful outcomes [6]. We present a case of locally invasive Fusarium infection to emphasize the critical role of early recognition and proper therapeutic intervention for a successful outcome.

## Case presentation

A 17-year-old female with acute lymphoblastic leukemia developed febrile neutropenia and necrotizing soft tissue infection of the left fourth toe at day 27 of consolidation chemotherapy. Tissue sample from the infected toe grew susceptible Pseudomonas aeruginosa for which she received piperacillin-tazobactam at a dose of 4.5 grams 6-hourly intravenously. Nevertheless, the infection progressed over the next 48 hours and spread locally to involve the dorsum of the left foot with signs of skin necrosis and with worsening pain.

**Figure 1:**
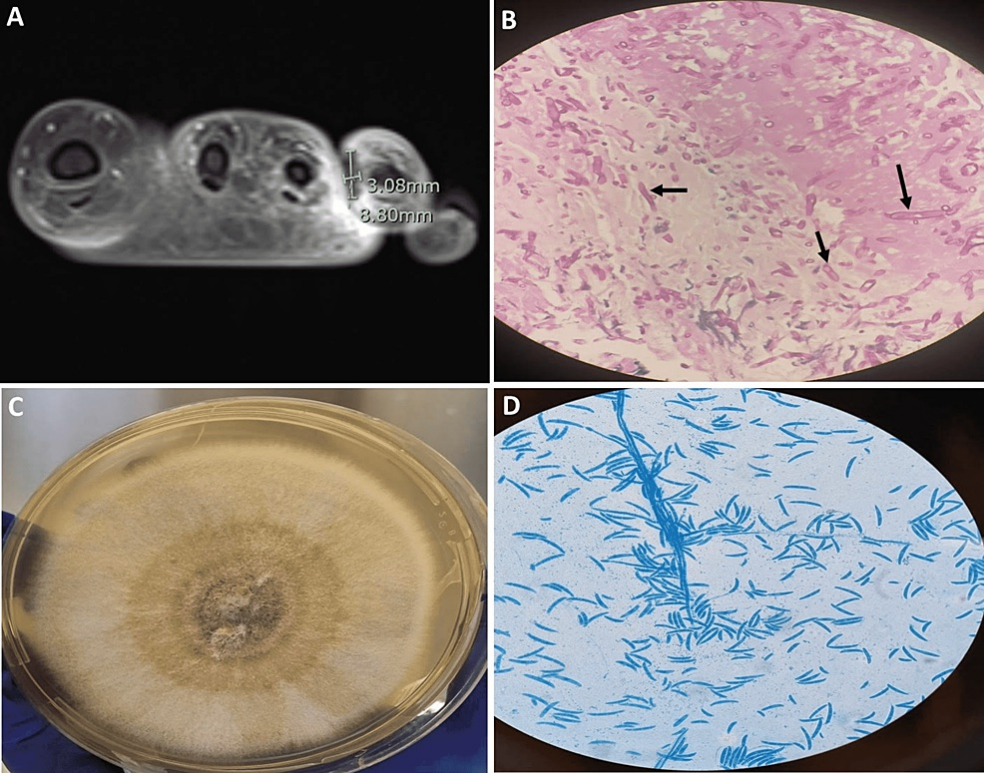
(A) Post-contrast MRI of the left foot showing focal area of hypoenhancement surrounded by hyperenhancement at the web space between the third and fourth toe; (B) Histopathologic examination of the left foot tissue with PAS stain showing angio-invasive septated hyphae (black arrows); (C) Fusarium solani isolated from Sabouraud dextrose agar at 30°C; (D) Microscopy examination of the fungal culture using lactophenol cotton blue stain showing canoe-shaped micro- and macroconidia, characteristic of Fusarium. PAS: Periodic Acid-Schiff

Physical examination revealed a conscious, ill-looking, and anxious patient with mild pallor. She was febrile (temperature: 38.90C), tachycardic (pulse rate: 109/min) and tachypneic (respiratory rate: 22/min). Her blood pressure was 119/73 mmHg. Detailed examination of the skin revealed no skin lesions. Local examination revealed a painful ulcerated swelling surrounded by minimal erythema over the left fourth toe and the web space between the third and fourth toe with marked swelling of the dorsum of the left foot. There was no definitive discharge. The rest of the examination was unremarkable. Magnetic resonance imaging (MRI) of the left foot showed a focal area of hypoenhancement surrounded by hyperenhancement at the web space between the third and fourth toe highly suggestive of angioinvasive mold infection (Figure 1A). Empiric initiation of liposomal amphotericin B (5 mg/kg) once daily combined with intravenous voriconazole (loading dose of 6 mg/kg for two doses followed by 4 mg/kg) twice daily was commenced. Furthermore, surgical exploration and debridement were undertaken. The frozen section and subsequent histopathological examination revealed septated hyphae with acute angle branching in a necrotic subcutaneous tissue and within the blood vessels (Figure 1B). The culture of the excised tissue grew Fusarium solani species (Figure 1C, 1D) which was further confirmed by matrix-assisted laser desorption/ionization-time of flight (MALDI-TOF) with a score of 2.28. Liposomal amphotericin and intravenous voriconazole were continued awaiting susceptibility testing in PHE mycology reference laboratory. During this period (approximately 10 days), near resolution of the local pathology is ascertained. The antifungal minimal inhibitory concentrations (MICs) for the isolate (Fusarium solani species) were as follows: amphotericin B 1.0 microgram/ml, isavuconazole 8.0 microgram/ml, posaconazole >16.0 microgram/ml, and voriconazole 16.0 microgram/ml, and were interpreted as resistant to all three azoles but susceptible to amphotericin. Subsequently, voriconazole was discontinued while liposomal amphotericin (5 mg/kg) once daily was continued for six weeks during which clinical, microbiological, and radiological cure was achieved. A secondary prophylaxis regimen of liposomal amphotericin B (7.5 mg/kg) once weekly was initiated with a plan to continue during consolidation chemotherapy.

## Discussion

Fusarium is an opportunistic hyalohyphomycotic fungus that can cause localized or disseminated infections depending on the host's immunity [7]. Fusariosis is an infection that affects plants, animals, and humans, and is caused by various fungi of the genus Fusarium [8]. There are at least 70 defined and well-described Fusarium species, however, only a few, in particular Fusarium oxysporum and Fusarium solani, are able to infect humans. Human infections with Fusarium are rare and sporadic but severe and often life-threatening [4].

Severely immunocompromised patients such as patients with severe chemotherapy-induced neutropenia are particularly vulnerable to infection with Fusarium, and clinical manifestations range from colonization to chronic localized lesions to acute invasive and/or disseminated diseases [7]. Our patient had localized albeit rapidly progressive extensive necrotizing soft tissue infection despite seemingly appropriate antipseudomonal antimicrobials. A high index of suspicion for invasive mold infection in our patient informed our clinical decision to empirically initiate antifungal therapy and to surgically intervene. Our experience with this case highlighted that early therapy of localized fusariosis is of paramount importance to prevent progression to a more aggressive or disseminated infection [9].

Diagnosis of invasive mold infections including fusariosis requires isolation and identification of the infecting mold. In our patient, histopathological examination of surgically debrided tissues demonstrated angioinvasion and provided a significant morphological description of the hyphae hence guided the initial antifungal therapy. However, microbiological identification of the infecting mold was necessary for targeted antifungal therapy. MALDI-TOF confirmed the isolate identification as Fusarium solani species. It is important to highlight that among all Fusarium species, Fusarium solani has the highest crude mortality rate of 67% [10].

European Confederation of Medical Mycology (ECMM) epidemiological survey on invasive infections due to Fusarium species in Europe observed a wide range of antifungal susceptibilities for Fusarium species [10]. Amphotericin B was the most potent antifungal in vitro, and itraconazole the least effective. Fusarium solani was found to be resistant to all three azoles tested [10]. This was consistent with the susceptibility testing result of this patient isolate demonstrating resistance to all three azoles tested. It is important to mention that there are no specific interpretative breakpoints for Fusarium species. Conversely, amphotericin B remains the active antifungal agent for Fusarium solani [11,12]. This was again demonstrated in this patient isolate with an MIC to amphotericin B of 1.0 microgram/ml. Therapy therefore should include amphotericin B with or without voriconazole and surgical debridement where possible [7].

## Conclusions

This case highlights the importance of clinical suspicion, early recognition, prompt diagnosis, and timely intervention to decrease the mortality rate of this severe infection. We propose that cutaneous lesions suspicious for fusariosis in the immunocompromised patient shall be quickly biopsied, histologically examined, cultured, and if feasible the isolate shall be subject to antifungal susceptibility testing. A high index of clinical suspicion is the first step towards attaining a successful outcome in this dreadful disease.
